# Reproductive toxicity after levetiracetam administration in male rats: Evidence for role of hormonal status and oxidative stress

**DOI:** 10.1371/journal.pone.0175990

**Published:** 2017-04-18

**Authors:** Merve Baysal, Sinem Ilgin, Gozde Kilic, Volkan Kilic, Seyda Ucarcan, Ozlem Atli

**Affiliations:** 1 Department of Pharmaceutical Toxicology, Faculty of Pharmacy, Anadolu University, Eskisehir, Turkey; 2 Department of Biology, Faculty of Science, Anadolu University, Eskisehir, Turkey; University of Hyderabad, INDIA

## Abstract

Levetiracetam (LEV) is an antiepileptic drug commonly used in the treatment of epilepsy because of its excellent safety profile in all age groups. It is remarkable that there are no studies evaluating the toxic effects of this drug on the male reproductive system, as it is commonly used in male patients of reproductive age. From this point of view, our aim was to evaluate the possible toxic effects of LEV on the male reproductive system. Therefore, LEV was administered to male rats orally at 50, 150, and 300 mg/kg for 70 consecutive days. At the end of this period, alterations to body and organ weights were calculated, and sperm concentration, motility, and morphology were investigated by a computer-assisted sperm analysis system. Sperm DNA damage was determined by comet assay and histopathological examination of the testes was carried out. Serum testosterone, follicle-stimulating hormone (FSH), and luteinizing hormone (LH) levels were measured by ELISAs to determine the effects of hormonal status, while glutathione, superoxide dismutase, catalase, and malondialdehyde levels in the testes were measured by colorimetric assay kits to determine the role of oxidative status in potential toxicity. According to the results, sperm quality was decreased by LEV treatment in a dose-dependent manner. LEV induced significant DNA damage in the 150 and 300 mg/kg LEV-administered groups. Histopathology of the testes showed that LEV resulted in testicular injury in the 300 mg/kg LEV-administered group. Serum testosterone, FSH, and LH levels were significantly decreased in the 300 mg/kg LEV-administered group. Glutathione, superoxide dismutase, and catalase levels were significantly decreased in all experimental groups while malondialdehyde levels were significantly increased in 150 and 300 mg/kg LEV-administered groups. According to these results, it was determined that LEV administration decreased sperm quality and it was alleged that hormonal alteration and oxidative stress are potential contributors to reproductive toxicity.

## Introduction

Reproductive toxicity has increased in recent years because of enhanced exposure to chemicals like environmental pollutants, industrial products, and pharmaceuticals [1]. Infertility is becoming the focus of interest among all reproductive toxic effects [2]. Results of epidemiological studies have suggested that approximately 50% of infertility cases are due to male-related factors. Pharmaceuticals are considered among the factors that may lead to infertility in males by decreasing the quality of semen [2, 3].

Reproductive toxicity occurs in epilepsy patients owing to the drugs used in treatment, as well as the disease itself [4, 5]. Reproductive dysfunction is observed in 22–67% of males with epilepsy. It is suggested that seizures and antiepileptic drugs change hormone levels and cause reproductive dysfunction by affecting the hypothalamic-pituitary-gonadal axis [6, 7]. Epilepsy affects approximately 50 million people worldwide [8]. It is therefore very important to determine the toxic effects of antiepileptic drugs that are frequently used in patients of reproductive age, during reproductive development and maturation processes [9].

Levetiracetam (LEV) is an antiepileptic drug that is frequently used in the treatment of epilepsy during childhood, adolescence, young adulthood, and adulthood periods [10, 11]. Although LEV is the first-line drug in the treatment of different types of epilepsy, there is insufficient data on the effects of this new-generation antiepileptic [4]. We aimed to assess the possible toxic effects of LEV on the male reproductive system in this study. Accordingly, sperm concentration, motility, morphology, and sperm DNA damage were determined and testis tissue was analyzed in a histopathological manner. In addition to this, serum testosterone, follicle-stimulating hormone (FSH), and luteinizing hormone (LH) levels were determined to evaluate the effect of hormonal status on possible toxicity. Glutathione (GSH), superoxide dismutase (SOD), catalase (CAT), and malondialdehyde (MDA) levels in the testis tissue were determined to evaluate the role of oxidative stress.

## Materials and methods

### Materials

LEV (Keppra^®^, UCB Pharma) tablets were used for experiments. FSH, LH, and testosterone levels in serum were determined by ELISA kits from Cusabio Biotech Co. Ltd., Hubei, P. R. C. GSH levels were measured with Cayman^®^ Glutathione Assay Kit, MI, USA. Also, MDA, CAT levels and SOD activity were determined by BioVision^®^ Lipid Peroxidation (MDA) Colorimetric/Fluorometric Assay Kit, CA, USA; BioVision^®^ Catalase Activity Colorimetric/Fluorometric Assay Kit, CA, USA; and BioVision^®^ Superoxide Dismutase (SOD) Activity Assay Kit, CA, USA; respectively.

### Animal model and experimental design

Male Wistar rats (12 weeks old, weighing 180–200 g) obtained from the Anadolu University Research Center for Animal Experiments were used for this study. Also this study was approved by the Local Ethical Committee on Animal Experimentation of Anadolu University, Eskisehir, Turkey (File Registration No: 2015–15). The rats were housed in a room at a controlled temperature (24°C) under a 12-h light/12-h dark cycle (lights on at 08:00 h) with free access to standard rat food and water. Animals were acclimatized to the laboratory environment for at least 48 h before the experimental session.

The rats were randomly divided into four groups, each group containing 10 rats. Administration duration of LEV was chosen according to the completion time of spermatogenesis and was in accordance with the guideline OECD 416: Guideline For Testing of Chemicals: Two-Generation Reproduction Toxicity Study [12], and doses of LEV were determined according to previous studies [13–15]. Also, the therapeutic doses of LEV were between 500 mg/day and 3000 mg/day [11] and the doses we have chosen were in accordance with the guidelines extrapolating human doses to animal doses [16]. LEV doses were administered at a volume of 1 mL/100 g by dissolving in distilled water. The rats were assigned randomly into the following treatment groups: *Control group (C)*, animals receiving distilled water by oral gavage for 70 days; *50 mg/kg LEV-administered group (LEV-50)*, animals receiving a 50 mg/kg dose of LEV by oral gavage for 70 days; *150 mg/kg LEV-administered group (LEV-150)*, animals receiving a 150 mg/kg dose of LEV by oral gavage for 70 days; *300 mg/kg LEV-administered group (LEV-300)*, animals receiving a 300 mg/kg dose of LEV by oral gavage for 70 days.

After LEV administration over 70 days, rats were anesthetized with urethane (1.5 g/kg, i.p.) [17]. Under general anesthesia, blood samples were collected via withdrawal of large amounts of blood from the right ventricle of the heart and rats were euthanized by cardiac puncture exsanguination.

Blood samples from the animals were kept at 2–8°C overnight, centrifuged at 1000 × g for 15 min, and the supernatant was collected. The serum samples were separated into 200 μL aliquots and stored at -20°C until levels of FSH, LH, and testosterone were measured.

Following the collection of blood samples, the testis and epididymis tissues were removed. The left testis and epididymis were washed with phosphate-buffered saline (PBS) and weighed. The left testis was then divided into equal parts and stored at -20°C until levels of oxidative stress parameters like MDA, GSH, SOD, and CAT were measured. The right testis was fixed for histopathological examination after washing with PBS. The cauda of the right epididymis was used to determine sperm parameters.

### Collection and evaluation of sperm samples

For sperm sampling, the rat cauda epididymis was immediately washed in PBS after removal, and placed in DMEM/Ham’s F-12 at 37°C. The cauda epididymis was cut into small pieces in a Petri dish containing the same medium to remove blood vessels and fat tissue. Half a centimeter of the cauda epididymis was transferred to another Petri dish containing 1 mL medium, and the sperm was allowed to swim up for 1 min.

Five microliters of the sperm suspension was loaded into a Leja slide (Leja Products BV, Nieuw Vennep, Netherlands). A 4× negative phase contrast objective combined with a phase contrast condenser was used to determine sperm motility and concentration via the Motility/Concentration module of the Sperm Class Analyzer^®^ version 5.4.0.1 software (Microptic S. L., Barcelona, Spain) at 25 frames/second. For motility analysis, 200 motile spermatozoa were analyzed as recommended [18, 19].

### Assessment of sperm morphology

Five microliters of the sperm suspension was placed on a slide and smeared with another slide. The sperm smears (three samples/animal) were totally dried at room temperature and stained with Spermblue^®^ (Microptic S. L., Barcelona, Spain) according to the method described by [20]. For morphology analysis, 200 spermatozoa were analyzed with the morphometry module of Sperm-Class Analyzer^®^ version 5.4.0.1 software (Microptic S. L., Barcelona, Spain). The machine was equipped with a Nikon Eclipse model 50i (Nikon Corporation, Tokyo, Japan) microscope with a 60× bright-field objective and a video camera (Basler, A78075gc, Germany). The abnormalities of the spermatozoa were evaluated according to the criteria from previous studies [21–25].

### Detection of sperm DNA damage by comet assay

For the comet assay, frosted slides were coated with 1% normal melting point agarose in Ca^2+^- and Mg^2+^-free PBS. Ten microliters fresh sperm sample (containing 1 x 10^5^ sperm/mL) was suspended in 75 μL of 1% low melting point agarose. This mixture was placed on a coated slide, covered with a lamella, and allowed to set at 4°C for 5 min. After solidification of the gel, the slide was immersed in lysis buffer (2.5 M NaCl, 100 mM EDTA, 10 mM Tris–HCl, pH 10.0 containing 1% triton X-100 added just before use, and 40 mM dithiothreitol) for 24 h at room temperature. After the first lysis step, proteinase K was added to the lysis solution (0.5 mg/mL) and a second lysis step was performed at 37°C for 24 h. At the end of these two lysis steps, all slides were washed three times with deionized water to remove salt and detergent from the micro gels. Following the washes, slides were placed on the platform of the electrophoresis apparatus and were allowed to equilibrate for 20 min with running buffer (500 mM NaCl, 100 mM Tris–HCl and 1 mM EDTA, pH 9) before electrophoresis (0.60 V/cm, 250 mA) for 30 min. The slides were then stained with SYBR Green I (1:10,000) for 1 h and covered with cover slips. DNA damage in spermatozoa was determined by measuring the tail moment via software BS 200 ProP, BAB Imaging System (Ankara, Turkey). One hundred randomly selected sperms were analyzed per sample [26].

### Histological analysis of testis tissue

Following the removal of the right testis, tissues were immediately sliced into small pieces (~2 mm^3^) and then fixed in paraformaldehyde (4%) in phosphate buffer pH 7.2. After dehydration of tissues in a graded series of alcohols, the samples were treated with a mixture of LR White resin (Electron Microscopy Sciences, PA, USA) and ethanol (2:1) (v:v) for 1 h at room temperature in order to improve infiltration. The samples were then embedded in LR White resin and sectioned at a thickness of 700 nm using a Leica EM UC7 ultramicrotome (Wetzlar, Germany). Semi-thin sections were stained with 1% toluidine blue/borax (pH 8.4) for 2 min and observed under a Leica DM 750 light microscope (Wetzlar, Germany) [27].

### Determination of serum FSH, LH, and testosterone levels

Blood samples from the animals were kept at 2–8°C overnight, centrifuged for 15 min (at 4°C and at 1000 x g) and the serum was separated. The hormonal analyses were performed using commercially available kits and in accordance with the manufacturer’s instructions.

### Determination of GSH and MDA levels in testis tissue

The left testis was divided into equal parts and stored at -20°C after freezing in liquid nitrogen. The GSH, CAT, SOD, and MDA levels in the testis were determined using commercially available kits and in accordance with the instructions of the manufacturer.

### Statistical analysis

All data are expressed as mean ± standard deviation. Statistical analyses of the groups were performed on the SigmaPlot v.10 package (Systat Software, CA, USA). All values were verified to be normally distributed. In the sperm comet assay, a Dunnett T3 test was performed as a post hoc test. In the other experiments, one-way ANOVA following a Tukey test as a post hoc test was performed. *P* < 0.05 was considered statistically significant.

## Results

### Effect of LEV administration on weight gains and relative testis/epididymis weights

Effects of LEV administration on weight were recorded as weight gain percentiles, and testis/epididymis weight changes were shown as relative testis/epididymis weights.

Relative testis/epididymis weights were expressed as g/ 100 g body weight. No differences were seen when comparing testis/epididymis weights in LEV-administered groups to the control group (Table 1).

**Table 1:** Effect of LEV administration on weight gain percentiles and relative organ weights.

Experimental Groups	Weight Gain %	Relative Testis Weights (g/100 g Body Weight)	Relative Epididymis Weights (g/100 g Body Weight)
C	71.35 ± 23.44	0.55 ± 0.10	0.26 ± 0.04
LEV-50	48.24 ± 16.15 (^a^)	0.52 ± 0.06	0.27 ± 0.04
LEV-150	47.18 ± 16.09 (^a^)	0.52 ± 0.09	0.27 ± 0.05
LEV-300	34.63 ± 12.41 (^aaa^)	0.51 ± 0.07	0.26 ± 0.03

C: Control group; LEV-50: 50 mg/kg levetiracetam administered group; LEV-150: 150 mg/kg levetiracetam administered group; LEV-300: 300 mg/kg levetiracetam administered group; Results are Mean ± SD, n = 10.

(^a^) Different from control group (*P* < 0.05),

(^aaa^) Different from control group (*P* < 0.001).

Weight gain percentiles in LEV-administered groups showed a significant decrease when compared with the control group. The most dramatic changes were observed in the LEV-300 group in comparison with the control group (Table 1).

### Effect of LEV administration on sperm parameters

#### Sperm concentration

As shown in Table 2, sperm concentration significantly decreased in the LEV-administered groups in comparison with the control group. While sperm concentration in the control group was 2.68 ± 0.88 x 10^6^/mL, sperm concentrations in the LEV-50, LEV-150, and LEV-300 groups were 0.83 ± 0.50 x 10^6^/mL, 0.82 ± 0.38 x 10^6^/mL, and 0.81 ± 0.37 x 10^6^/mL, respectively.

**Table 2:** Effect of LEV administration on sperm concentration and motility.

Experimental Groups	Sperm Concentrations (10^6^/mL)	Sperm Motility (%)
C	2.68 ± 0.88	84.54 ± 9.08
LEV-50	0.84 ± 0.22 (^a^)	78.83 ± 8.16
LEV-150	0.82 ± 0.38 (^a^)	80.49 ±3.89
LEV-300	0.81 ± 0.37 (^a^)	69.78 ± 13.02 (^a^)

C: Control group; LEV-50: 50 mg/kg levetiracetam administered group; LEV-150: 150 mg/kg levetiracetam administered group; LEV-300: 300 mg/kg levetiracetam administered group; Results are Mean ± SD, n = 10.

(^a^) Different from control group (*P* < 0.05).

#### Sperm motility

The percentage of motile sperm in the control group was 84.54 ± 9.08%, while it was determined as 78.83 ± 8.16%, 80.49 ± 3.89%, and 69.78 ± 13.02% in the LEV-50, LEV-150, and LEV-300 groups, respectively. According to these results, a significant decrease was observed in the LEV-300 group compared with the control group. No significant differences were observed among LEV-administered groups in terms of sperm motility (Table 2).

#### Sperm morphology

The percentage of abnormal sperm in the LEV-150 and LEV-300 groups significantly increased when compared to the control group. Among LEV-administered groups, the LEV-300 group showed a significantly higher abnormal sperm percentage when compared to the LEV-50 group (Table 3).

**Table 3:** Effect of LEV administration on sperm morphology.

Experimental Groups	Abnormal Sperm (%)	Head Abnormality (%)	Tail Abnormality (%)	Multiple Abnormality (%)
C	24.85 ± 4.88	8.75 ± 3.22	12.4 ± 3.74	3.7 ± 1.58
LEV-50	38.7 ± 2.98	13.55 ± 3.24	18.3 ± 2.09	6.5 ± 2.04
LEV-150	46.3 ± 7.02 (^a^)	13.3 ± 3.14	25.85 ± 3.34 (^a^)	7.15 ± 2.75 (^a^)
LEV-300	59.25 ± 7.21 (^aaa^^,^^b^)	15.05 ± 3.59 (^a^)	35.5 ± 4.47 (^aaa^^,^^b^)	8.7 ± 3.72 (^a^)

C: Control group; LEV-50: 50 mg/kg levetiracetam administered group; LEV-150: 150 mg/kg levetiracetam administered group; LEV-300: 300 mg/kg levetiracetam administered group; Results are Mean ± SD, n = 10.

(^a^) Different from control group (*P* < 0.05),

(^aaa^) Different from control group (*P* < 0.001),

(^b^) Different from LEV-50 (*P* < 0.05).

**Fig 1:**
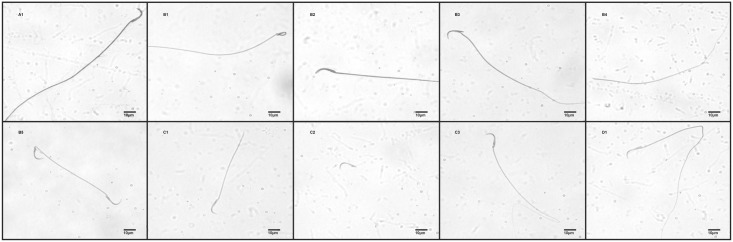
Sperm morphology observed under 60 x magnification following LEV administration. A1: Normal; B: Head abnormalities; B1: Amorphous; B2: Banana; B3: Bent neck; B4: Headless; B5: Two headed; C: Tail abnormalities; C1: Broken tail; C2: Detached; C3: Bent tail; D: Multiple abnormalities; D1: Banana + Bent tail.

The LEV-300 group showed a significant increase in head abnormalities when compared with the control group. According to percentage of tail abnormalities, significant increases were observed in the LEV-150 and LEV-300 groups when compared with the control group. Among LEV-administered groups, the LEV-300 group showed a significantly higher percentage of tail abnormalities when compared to the LEV-50 group. Percentage of multiple abnormalities in the LEV-150 and LEV-300 groups were significantly higher than the control group (Fig 1, Table 3).

### Sperm DNA damage

**Fig 2:**
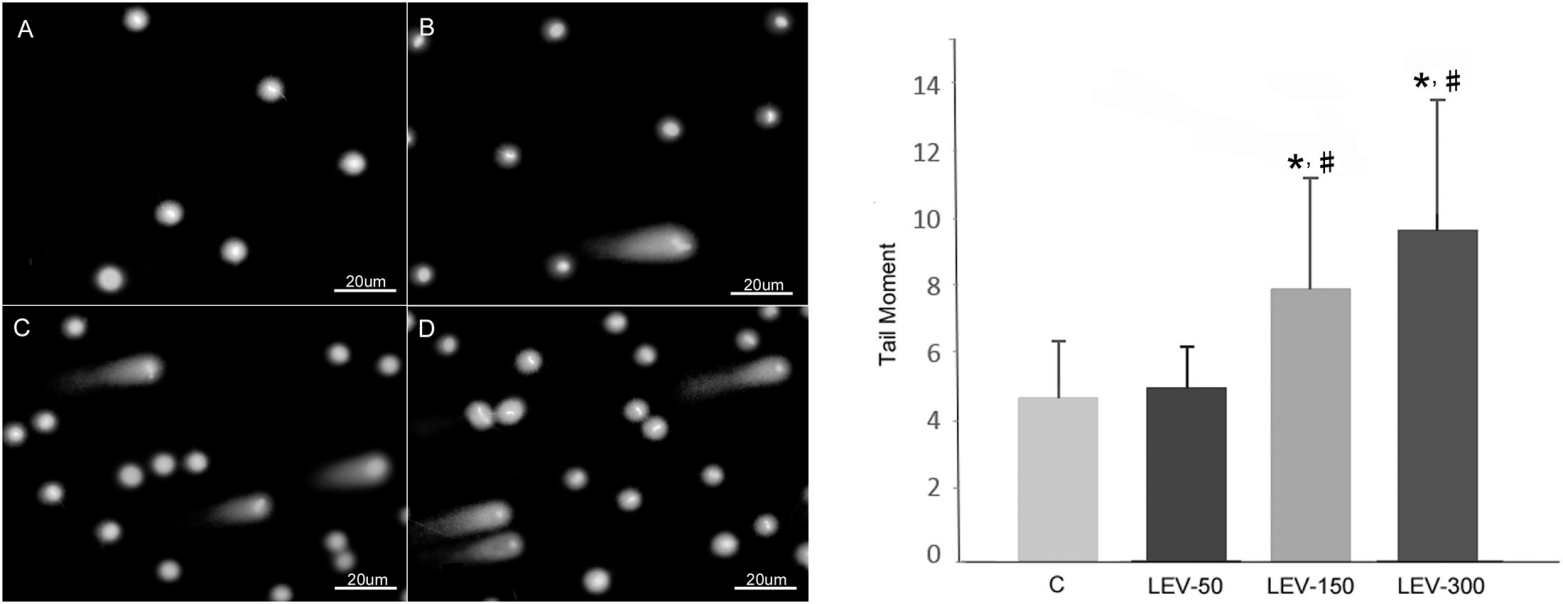
Effect of LEV on sperm DNA damage. (A) Sperm comet assay photo of control group, (B) Sperm comet assay photo of LEV-50, (C) Sperm comet assay photo of LEV-150, (D) Sperm comet assay photo of LEV-300; Tail moment graph: C: Control group; LEV-50: 50 mg/kg LEV administered group, LEV-150: 150 mg/kg LEV administered group, LEV-300: 300 mg/kg LEV administered group. * Significant differences when compared with control group (*P* < 0.05); ^#^ Significant differences when compared with LEV-50 group (*P* < 0.05).

The results expressed as tail moment (extent tail moment: tail length x tail%DNA / 100) for sperm exposed to different doses of LEV and for control group are shown in Fig 2. Exposure to 150 and 300 mg/kg of LEV increased the tail moment by about 67.35% and 104.75% (about two-fold), respectively, when compared to the control. Tail moment also increased in these groups by 58.20% and 93.55%, respectively, when compared to the LEV-50 group.

### Histological analysis of testis tissue

**Fig 3:**
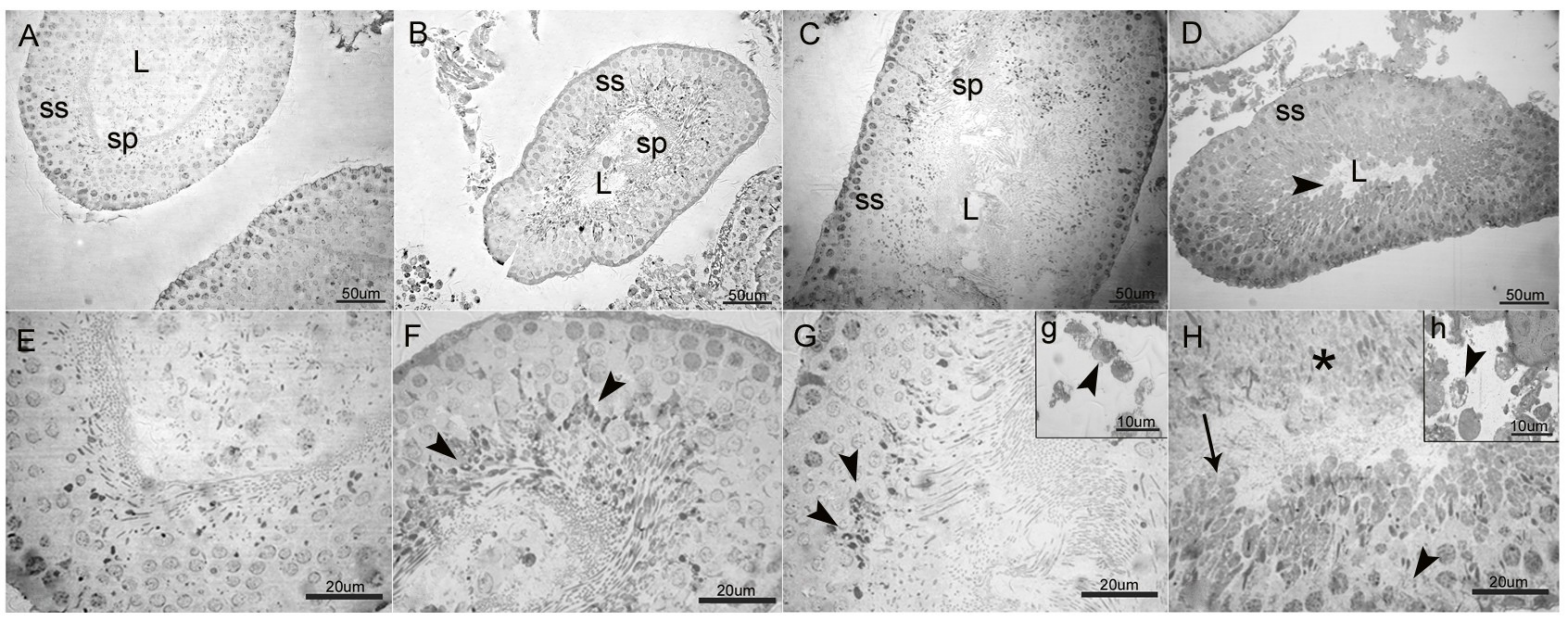
General view of the seminiferous tubules (A-D) and high magnification of germinal epithelium (E-H) in LEV administered groups and control. (A-D; L: lumen; ss: spermatogenic series; sp: spermatozoa, scale bar: 50 μm). A: Normal architecture of the seminiferous tubule associated with complete spermatogenic series (ss) in control. B & C: Relatively preserved structure of seminiferous tubules and considerable number of spermatozoa in the tubular lumen in 50 mg/kg and 150 mg/kg LEV administered groups, respectively. D: Tubular degeneration with disorganisation of spermatogenic series and markedly decreased number of sperms (►) in the tubular lumen in 300 mg/kg LEV administered group. (E-H; scale bar: 20 μm). E: Intact layers of spermatogenic series in control. F: Slight vacuolation at late stage spermatids (►) in 50 mg/kg LEV administered group. G: Increased vacuolation and slight degeneration at late stage spermatids (►) in 150 mg/kg LEV administered group. H: Loss of cellular architecture in spermatogenic series (►), swelling, vacuolation (→) and necrosis (*) of the spermatids in 300 mg/kg LEV administered group. g & h: High magnification of Leydig cells (Scale bar: 10 μm). g: Vacuolation in Leydig cells (►) in 150 mg/kg LEV administered group. h: Increased vacuolation and cellular degeneration in Leydig cells (►) in 300 mg/kg LEV-administered group.

Control group animals showed normal architecture of the seminiferous tubules with regularly arranged rows and complete set of germinal epithelium (Fig 3A and 3E). Tubular degeneration was not observed in the LEV-50 group; however, there was a slight vacuolation in late stage spermatids (Fig 3B and 3F). In the LEV-150 group, late stage spermatids and mature spermatozoa were relatively fewer and showed increased vacuolation as was also observed in Leydig cells (Fig 3C, 3G and 3g). LEV (300 mg/kg) administration dramatically increased the lesions which resulted in tubular degeneration and loss of cellular architecture in spermatogenic series. Late stage spermatids and many early spermatids were swollen and vacuolated, and necrosis was observed in some parts of the seminiferous tubules. Very few mature spermatids were observed in the tubular lumen (Fig 3D, 3H and 3h). In accordance with these findings, Johnsen’s scoring of the 50 mg/kg LEV-administered group was similar to that of the control (9.49 ±0.22 and 9.68 ±0.16 respectively). However, 150 and 300 mg/kg LEV administration resulted in a significant decrease in these scores, with 8.93 ±0.34 in LEV-150 group and 7.51 ±0.31 in LEV-300 group, indicating the increase in the impairment of spermatogenesis at higher doses (Table 4).

**Table 4:** Johnsen’s scores and semi-quantitative comparison of pathology at the cellular level.

Experimental Groups	Johnsen’s score	Spermatogonial swelling	Cytoplasmic vacuolation	Deformation of celluar architecture
C	9.677 ± 0.160	-	-	-
LEV-50	9.485 ± 0.215	-	+	-
LEV-150	8.934 ± 0.343 (^a^)	-	++	+
LEV-300	7.514 ± 0.309 (^a^)	++	+++	+++

C: Control group; LEV-50: 50 mg/kg levetiracetam administered group; LEV-150: 150 mg/kg levetiracetam administered group; LEV-300: 300 mg/kg levetiracetam administered group; Results are Mean ± SD, n = 160.

(^a^) Different from control group (*P* < 0.05).

### Effect of LEV administration on serum hormone levels

When serum FSH levels were compared between groups, LEV-300 group showed a significant decrease in comparison with the control group. Additionally, FSH levels of the LEV-300 group were significantly reduced compared to the LEV-50 and LEV-150 groups (Table 5).

**Table 5:** Effect of LEV administration on reproductive hormones FSH, LH, and testosterone.

Experimental Groups	FSH (IU/L)	LH (mg/dL)	Testosterone (IU/L)
C	12.63 ± 2.73	18.16 ± 3.73	4.87 ± 2.6
LEV-50	11.26 ± 1.76	16.14 ± 2.91	3.05 ± 1.85
LEV-150	10.44 ± 3.55	15.70 ± 3.05	3.44 ± 1.39
LEV-300	6.34 ± 1.77 (^a^^,^^b^^,^^c^)	11.13 ± 2.90 (^a^^,^^b^^,^^c^)	2.48 ± 1.26 (^a^)

C: Control group; LEV-50: 50 mg/kg levetiracetam administered group; LEV-150: 150 mg/kg levetiracetam administered group; LEV-300: 300 mg/kg levetiracetam administered group; Results are Mean ± SD, n = 10.

(^a^) Different from control group (*P* < 0.05),

(^b^) Different from LEV-50 (*P* < 0.05),

(^c^) Different from LEV-150 (*P* < 0.05).

According to the serum levels of LH in our study, serum LH level of the LEV-300 group decreased significantly compared to the control group. A significant decrease was also observed in the LEV-300 group compared to the LEV-50 and LEV-150 groups (Table 5).

When serum testosterone levels were compared across the different groups, testosterone levels in the LEV-300 group were lower than the control group. The results were not significantly different among LEV-administered groups regarding serum testosterone level (Table 5).

### Effect of LEV administration on oxidative stress parameters in testis tissue

SOD activity in the LEV-150 and LEV-300 groups was significantly decreased compared to the control group. The LEV-300 group showed significant differences compared with the LEV-50 and LEV-150 groups (Table 6).

**Table 6:** Effect of LEV administration on oxidative stress parameters SOD activity, CAT, GSH, and MDA levels in testis tissue.

Experimental Groups	SOD Activity (%)	CAT (mU/mL)	GSH (μm)	MDA (Nmol/g Tissue)
C	97.94 ± 1.13	60.47 ± 12.97	217.48 ± 15.95	0.56 ± 0.16
LEV-50	95.5 ± 2.15 (^a^)	37.65 ± 12.13 (^a^)	120.99 ± 7.92 (^a^)	0.82 ± 0.43
LEV-150	92.16 ± 4.17 (^a^)	35.94 ± 7.23 (^a^)	101.07 ± 11.62 (^a^)	0.94 ± 0.49 (^a^)
LEV-300	86.82 ± 6.67 (^a^^,^^b^^,^^c^)	33.56 ± 4.91 (^a^)	92.64 ± 10.09 (^aaa^^,^^b^)	1.09 ± 0.48 (^a^)

C: Control group; LEV-50: 50 mg/kg levetiracetam administered group; LEV-150: 150 mg/kg levetiracetam administered group; LEV-300: 300 mg/kg levetiracetam administered group; Results are Mean ± SD, n = 10.

(^a^) Different from control group (*P* < 0.05),

(^aaa^) Different from control group (*P* < 0.001),

(^b^) Different from LEV-50 (*P* < 0.05),

(^c^) Different from LEV-150 (*P* < 0.05).

CAT levels were decreased in all LEV-administered groups compared with the control group. There were no significant differences in CAT levels among the LEV-administered groups (Table 6).

According to GSH levels in testis tissue, GSH levels reduced in LEV-administered groups compared to the control group. In addition, GSH levels in the LEV-300 group were significantly lower than the LEV-50 group (Table 6).

MDA levels were increased significantly in all LEV-administered groups compared with the control group. No difference was observed among LEV-administered groups regarding MDA levels (Table 6).

## Discussion

In the present study, we evaluated the reproductive toxicity of LEV in male rats, independently of other risk factors related to infertility, and with repeated pharmacological dosing. The results showed that LEV decreased sperm concentration, motility, normal sperm morphology, increased sperm DNA damage, and induced histopathological changes in testicular tissue in a dose-dependent manner. These toxic effects induced by LEV have been accompanied by induced oxidative stress in testicular tissue and the alteration of serum hormon levels, which play a role in the spermatogenesis process.

Body and organ weights are sensitive biomarkers that are used to determine toxicity related to chemical exposure [28]. Changes in the weight of related tissues reflect the disruptions in the functions of the male reproductive system [29]. In our study, significant differences in relative testis and epididymis weights among groups after administration of LEV were not observed. At this point, it must be emphasized that relative weight differences may escape notice because of factors like edemas occurring in testis and epididymis tissue, or congestion of efferent channels [30]. Another finding regarding weight changes in our study was the dose-dependent decrease in weight change % after administration of LEV. The effect of antiepileptic drugs on weight loss has been studied for many years [31, 32]. Significant weight loss has been reported after LEV treatment and it is suggested that LEV may cause weight loss at levels that disrupt quality of life in a dose-dependent manner [33].

Sperm concentration, motility, and morphology are biomarkers used to evaluate semen quality and determine reproductive system toxicity [34]. In our study, dose-dependent decreases in sperm concentration in rats have been observed after administration of LEV. In support of this finding, decreasing spermatid counts in tubular lumen were seen in histopathological analyses. Sperm motility was significantly decreased in our high-dose group. Sperm motility is needed for the sperm to reach the cervix and fallopian tubes, and unite with the oocyte. Thus, a decrease in sperm motility is considered an important factor of chemical-induced infertility [29, 35]. Sperm morphology development is another important parameter in determining semen quality, and continues into the late stages of spermatogenesis [34]. Therefore, sperm morphology is a result of the maturation process of sperm in the testes, and is considered as an important biomarker to evaluate testicular functions [29]. Regulatory authorities like EPA, FDA, OECD, WHO, and ICH stress the importance of the abnormalities in the sperm head in the study of male reproductive toxicity [26]. In addition to this, abnormalities in sperm midpiece and tail may also be a factor contributing to infertility [20]. Sperm tail abnormalities, especially twisted tail and bent/spiral tail abnormalities, are considered to be related to infertility [4]. In our study, abnormalities in sperm morphology were detected related to LEV treatment, particularly in the LEV-150 and LEV-300 groups. Head abnormalities that were considered as important biomarkers regarding infertility were observed in the LEV-300 group and tail abnomalities were observed in both LEV-150 and LEV-300 groups. Even though data in regarding LEV’s effects on sperm parameters are insufficient, studies have shown that other antiepileptic drugs cause decreases in sperm concentration, motility, and normal sperm morphology [4, 36–38]. Therefore, although LEV is considered safer than other antiepileptic drugs because of its different mechanism of action, its potentially adverse effects on semen quality are similar to other antiepileptic drugs and should not be ignored. In light of these results, it can be suggested that LEV treatment may negatively affect fertility by decreasing semen quality in males.

FSH, LH, and testosterone have roles in the maintenance of male reproductive functions and therefore determining levels of these hormones is important in reproductive toxicity studies [39]. It is known that LH and FSH are secreted under the control of hypothalamic gonadotropin-releasing hormone from the anterior pituitary. LH stimulates the release of testosterone from Leydig cells, and testosterone is necessary for the development of secondary sexual characteristics, spermatogenesis, and the transfer/storage of spermatozoa in the epididymis. FSH regulates the production of spermatozoa in Sertoli cells [40]. The hypothalamic-pituitary-gonadal axis can be affected by many factors. Chemicals including drugs can decrease fertility and even lead to infertility in both males and females by disrupting the normal function of this axis [41]. It has been shown that antiepileptic drugs also influence hypothalamic-pituitary-gonadal axis and cause reproductive dysfunction [6, 36–38, 42–44]. In our study, decreased serum FSH, LH, and testosterone levels were observed in our high-dose group. Studies showed that LH and FSH levels were not altered [44, 45], while testosterone levels were increased [44–46] in male epileptic patients due to LEV treatment. Epilepsy treatment with LEV in prepubertal children did not cause a meaningful change in hormone levels [47]. A decrease in serum LH levels can directly decrease the testosterone production from Leydig cells [40]. Additionally, a decrease in testosterone levels is considered to be related to the severity of spermatogenic cell damage and disruptions in spermatogenesis, and can therefore cause reproductive dysfunction [40, 41]. Histopathological findings in our study also indicated that vacuolization that was mildly observed in our low-dose group was followed by cellular swelling, spermatid damage, degeneration, and necrosis becoming more pronounced in a dose-dependent manner. Decreased testosterone levels can also be a cause of decrease in semen quality [29]. Therefore, decreased semen quality in our LEV-administered groups could be considered as a consequence of decreased testosterone levels. The basis of the effective mechanism which differentiates LEV from other antiepileptic drugs is that it binds to the synaptic vesicle glycoprotein 2A (SV2A). SV2A is distributed in endocrine tissues as well as in the central nervous system. Previous studies stressed that LEV’s endocrine effects can be mediated by this protein [44, 46]. Additionally, it must be emphasized that antiepileptic drugs cause reproductive and sexual disorders by modulating serotonergic transmission [6]. It has been proposed that LEV can lead to sexual dysfunctions by disrupting dopamine/serotonin balance [48]. Increased serotonin levels may disrupt the feedback mechanism of the hypothalamic-pituitary-gonadal axis [49]. Therefore, the possible effects of LEV on the serotonergic pathway may contribute to changes in FSH, LH, and testosterone levels.

It has been suggested that oxidative stress is an important factor in the decrease of semen quality, and male infertility [50, 51]. Oxidative stress causes a decrease in intracellular ATP levels, release of apoptogenic factors (pro-caspase cytochrome C, apoptosis-inducing factors) into the cytosol as a result of disruption of the mitochondrial membrane, enzyme dysfunction, disruption of protein phosphorylation, increase in membrane permeability, and formation of spermicidal products, and therefore decreases semen quality [50]. The particularly high polyunsaturated fatty acids content of sperm membrane makes it sensitive to oxidative stress [34, 51]. Additionally, insufficiency of the antioxidant defense mechanism in sperm contributes to this vulnerability [52]. In our study, reduced SOD, CAT, and GSH levels and increased MDA levels were detected after administration of LEV indicating that this drug resulted in the induction of oxidative stress in testicular tissue. Studies with opposing results have shown that administration of LEV had antioxidant effects in pathologies where oxidative stress is induced [53–57]. However, in another study, an increase in oxidative stress in the brain tissue of rats after administration of LEV was detected, similarly to that in our study [58]. In support of LEV-induced oxidative stress a previous study has shown decreases in antioxidant parameters and increases in oxidant parameters due to LEV treatment in epileptic patients [59]. Hence, the detected decrease in semen quality in the LEV-administered groups in our study can be consequent to the oxidative stress induced by LEV in testicular tissue.

Sperm DNA integrity is a sign of the reproductive capability of the sperm [60]. Therefore, the DNA’s structural integrity needs to be investigated in order to evaluate sperm function and its structural changes [34]. The neutral comet assay is easier, more cost-effective, and more frequently used than other methods in determining DNA damage in human sperm, as it is considered as a more sensitive method in detecting double-strand breaks [34, 61]. The most commonly used parameters in the quantitation of DNA damage by comet method are tail length, DNA percentage in tail, and tail moment. It is argued that tail length can be used to detect the initial damage caused when exposed to a genotoxic agent, while tail moment and DNA percentage in tail can be used to detect the severity of DNA damage [62, 63]. However, many studies concluded that tail moment is a better measure to assess DNA damage [64–67]. According to the tail moment data from our study, an increase in sperm DNA damage in the LEV-administered groups was detected by neutral comet assay. Another study which used comet assay showed that administration of LEV in female rats during their 5^th^-18^th^ days of pregnancy resulted in an increase of DNA damage in brain and liver tissues of both female rats and their fetuses [68]. It is argued that ROS may cause sperm DNA damage and lead to the development of infertility in males [34]. Insufficiency of sperm DNA repair mechanisms increases the sensitivity of sperm DNA to oxidative stress even further [52]. In support of these findings, the most important evidence that suggests oxidative DNA damage in sperm is related to male infertility, is high levels of 8-hydroxydeoxyguanosine, which is a product of oxidative DNA observed in infertile males [34]. It is also known that LEV causes histone modifications in DNA by inhibiting the histone deacetylase enzyme, and this results in chromatin decondensation [69, 70]. Thus, LEV-induced DNA damage observed in our study may be the result of oxidative stress, and DNA histone modification by LEV may be a factor promoting this situation. Furthermore, sperm head morphology is considered to be an indirect sign of chemically induced mutagenic effects on sperm DNA. In a previous study, a positive correlation between sperm head abnormalities and sperm DNA damage was detected, and it was argued that defects in sperm head morphology resulted from damages in the genetic material of the sperm [26]. In our study, sperm head abnormalities increased by administration of LEV can be considered as a sign of LEV-induced DNA damage.

According to the results of our study, LEV, which is a first-line drug in the treatment of epilepsy, adversely affected sperm parameters by reducing sperm concentration and motility, increasing abnormal sperm morphology and sperm DNA damage, and caused damage in the testicular structure of male rats. It is suggested that the mechanism of LEV toxicity in the male reproductive system arises from the promotion of oxidative stress and alterations in hormonal status. However, there is still a need for additional human data to investigate the potential risk of LEV on reproductive function in male patients. Further studies may also be conducted to investigate the effects of LEV on fertilization. It is also emphasized that investigating reproductive toxicity and fertility levels in patients using LEV through clinical studies is necessary. On the other hand, we suggest that sperm DNA damage and oxidative status in semen can be determined in addition to traditional sperm parameters.

## References

[1] Hoyer PB. Reproductive toxicology: current and future directions. Biochem Pharmacol. 2001 12 15;62(12):1557–64. 11755108 10.1016/s0006-2952(01)00814-0

[2] Liu L, Bao H, Liu F, Zhang J, Shen H. Phthalates exposure of Chinese reproductive age couples and its effect on male semen quality, a primary study. Environ Int. 2012 7;42:78–83. 10.1016/j.envint.2011.04.005 21524797

[3] McPhee SJ. Disorders of the Male Reproductive Tract In: McPhee SJ, Lingappa WR, Ganong WF, Lange JD editors. Pathophysiology of Disease-An Introduction to Clinical Medicine 3^rd^ ed New York: McGraw-Hill; 2000 pp. 556–76.

[4] Svalheim S, Sveberg L, Mochol M, Taubøll E. Interactions between antiepileptic drugs and hormones. Seizure. 2015 5;28:12–7. 10.1016/j.seizure.2015.02.022 25797888

[5] Verrotti A, Loiacono G, Laus M, Coppola G, Chiarelli F, Tiboni GM. Hormonal and reproductive disturbances in epileptic male patients: emerging issues. Reprod Toxicol. 2011 5;31(4):519–27. 10.1016/j.reprotox.2011.02.002 21338669

[6] Calabrò RS, Marino S, Bramanti P. Sexual and reproductive dysfunction associated with antiepileptic drug use in men with epilepsy. Expert Rev Neurother. 2011 6;11(6):887–95. 10.1586/ern.11.58 21651335

[7] Montouris G, Morris GL 3rd. Reproductive and sexual dysfunction in men with epilepsy. Epilepsy Behav. 2005 12;7(2):S7–14. 16243004 10.1016/j.yebeh.2005.08.026

[8] WHO Fact Sheets 2016 [Internet]. World Health Organization. Epilepsy: Key facts [cited 2016 Jun 7]. http://www.who.int/mediacentre/factsheets/fs999/en/

[9] Kozłowski P, Czępińska-Ćwik W, Kozłowska M, Kozłowska K. Levetiracetam-epilepsy treatment, pharmacokinetics, mechanism of action, interaction and toxicity. Journal of Education, Health and Sport, 2015;5(4),143–50.

[10] Kwong KL, Tsui KW, Wu SP, Yung A, Yau E, Eva F et al Utilization of antiepileptic drugs in Hong Kong children. Pediatr Neurol. 2012 5;46(5):281–6. 10.1016/j.pediatrneurol.2012.02.019 22520348

[11] Lyseng-Williamson KA. Levetiracetam: a review of its use in epilepsy. Drugs. 2011 3 5;71(4):489–514. 10.2165/11204490-000000000-00000 21395360

[12] OECD [Internet]. Guideline For Testing of Chemicals Two-Generation Reproduction Toxicity Study Guideline 416 dated January 2001 [cited 2015 May 27]. http://www.oecdilibrary.org/docserver/download/9741601e.pdf?expires=1394537191&id=id&accname=guest&checksum=4BD261B2270C036B2009759CF5AE3A1C

[13] Kitano Y, Komiyama C, Makino M, Takasuna K, Satoh H, Aoki T et al Anticonvulsant and neuroprotective effects of the novel nootropic agent nefiracetam on kainic acid-induced seizures in rats. Brain Res. 2005 9 28;1057(1–2):168–76. 16122714 10.1016/j.brainres.2005.07.052

[14] Margineanu DG, Matagne A, Kaminski RM, Klitgaard H. Effects of chronic treatment with levetiracetam on hippocampal field responses after pilocarpine-induced status epilepticus in rats. Brain Res Bull. 2008 11 25;77(5):282–5. 10.1016/j.brainresbull.2008.07.006 18722515

[15] Svalheim S, Taubøll E, Surdova K, Ormel L, Dahl E, Aleksandersen M et al Long-term levetiracetam treatment affects reproductive endocrine function in female Wistar rats. Seizure. 2008 3;17(2):203–9. 18155931 10.1016/j.seizure.2007.11.018

[16] CDER [Internet]. U.S. Department of Health and Human Services Food and Drug Administration Center for Drug Evaluation and Research (CDER): Estimating the Maximum Safe Starting Dose in Initial Clinical Trials for Therapeutics in Adult Healthy Volunteers [cited 2017 Feb 6]. http://www.fda.gov/downloads/drugs/guidances/ucm078932.pdf

[17] Takeuchi K, Takayama S, Hashimoto E, Itayama M, Amagase K, Izuhara C. Effect of rebamipide on gastric bleeding and ulcerogenic responses induced by aspirin plus clopidogrel under stimulation of acid secretion in rats. J Gastroenterol Hepatol. 2014 12;29(4):37–46. 25521732 10.1111/jgh.12774

[18] WHO [Internet]. WHO laboratory manual for the examination of human semen and sperm cervical mucus interaction 1999 [cited 2015 May 27]. http://apps.who.int/iris/bitstream/10665/44261/1/9789241547789_eng.pdf

[19] Maree L, van der Horst G. Quantification and identification of sperm subpopulations using computer-aided sperm analysis and species-specific cut-off values for swimming speed. Biotech Histochem. 2013 5;88(3–4):181–93. 10.3109/10520295.2012.757366 23331185

[20] van der Horst G, Maree L. SpermBlue: a new universal stain for human and animal sperm which is also amenable to automated sperm morphology analysis. Biotech Histochem. 2009 12;84(6):299–308. 10.3109/10520290902984274 19488904

[21] Filler R. Methods for evaluation of rats epididymal sperm morphology In: Chapin RE, Heindel JH editors. Male reproductive toxicology. California: Academic Press; 1993 pp. 334–43.

[22] Gromadzka-Ostrowska J, Dziendzikowska K, Lankoff A, Dobrzyńska M, Instanes C, Brunborg G, Gajowik A, Radzikowska J, Wojewódzka M, Kruszewski M. Silver nanoparticles effects on epididymal sperm in rats. Toxicol Lett. 2012 11 15;214(3):251–8. 10.1016/j.toxlet.2012.08.028 22982066

[23] Martinez CS, Torres JG, Peçanha FM, Anselmo-Franci JA, Vassallo DV, Salaices M et al 60-Day chronic exposure to low concentrations of HgCl_2_ impairs sperm quality: hormonal imbalance and oxidative stress as potential routes for reproductive dysfunction in rats. PLoS One. 2014 11 4;9(11):e111202 10.1371/journal.pone.0111202 25368988 PMC4219708

[24] Mori K, Kaido M, Fujishiro K, Inoue N, Koide O, Hori H et al Dose dependent effects of inhaled ethylene oxide on spermatogenesis in rats. Br J Ind Med. 1991 4;48(4):270–4. 2025594 10.1136/oem.48.4.270 PMC1035369

[25] Narayana K, D'Souza UJ, Seetharama Rao KP. Ribavirin-induced sperm shape abnormalities in Wistar rat. Mutat Res. 2002 1 15;513(1–2):193–6. 11719104 10.1016/s1383-5718(01)00308-4

[26] Trivedi PP, Kushwaha S, Tripathi DN, Jena GB. Evaluation of male germ cell toxicity in rats: correlation between sperm head morphology and sperm comet assay. Mutat Res. 2010 12 21;703(2):115–21. 10.1016/j.mrgentox.2010.08.005 20713175

[27] Bancroft JD, Gamble M. Theory and Practice of Histological Techniques. 6th ed Edinburgh: E.P. Churchill Livingstone; 2002.

[28] Kong L, Tang M, Zhang T, Wang D, Hu K, Lu W et al Nickel nanoparticles exposure and reproductive toxicity in healthy adult rats. Int J Mol Sci. 2014 11 17;15(11):21253–69. 10.3390/ijms151121253 25407529 PMC4264223

[29] Jayachandra S, AnnGie N. Possible toxic effect of antihypertensive drug olmesartan on male reproductive system of rat. Int J Basic Clin Pharmacol. 2013;2(1),83–8.

[30] Kimmel GL, Clegg ED, Crisp TM. Reproductive Toxicity Testing: A Risk Assessment Perspective Witorsch RJ editor. In: Reproductive Toxicology 2^nd^ ed New York: Raven Press, Ltd; 1995 pp. 75–99.

[31] Antel J, Hebebrand J. Weight-Reducing Side Effects of the Antiepileptic Agents Topiramate and Zonisamide, In: Joost HG. editor. Appetite control. Berlin: Springer; 2012 pp. 433–7. 10.1007/978-3-642-24716-3_20 22249827

[32] Ben-Menachem E. Weight issues for people with epilepsy—a review. Epilepsia. 2007;48(9):42–5. 10.1111/j.1528-1167.2007.01402.x 18047602

[33] Gelisse P, Juntas-Morales R, Genton P, Hillaire-Buys D, Diaz O, Coubes P et al Dramatic weight loss with levetiracetam. Epilepsia. 2008 2;49(2):308–15. 10.1111/j.1528-1167.2007.01273.x 17825078

[34] Ong CN, Shen HM, Chia SE. Biomarkers for male reproductive health hazards: are they available?. Toxicol Lett. 2002 8 5;134(1–3):17–30. 12191857 10.1016/s0378-4274(02)00159-5

[35] Toman R, Hluchy S, Massanyi R, Lukac N, Adamkovicova M, Cabaj M et al Selenium and Cadmium Tissue Concentrations and the CASA Sperm Motility Analysis after Administration to Rats. Am J Anim Vet Sci. 2014;9(4):194–202.

[36] Daoud AS, Bataineh H, Otoom S, Abdul-Zahra E. The effect of Vigabatrin, Lamotrigine and Gabapentin on the fertility, weights, sex hormones and biochemical profiles of male rats. Neuro Endocrinol Lett. 2004 6;25(3):178–83. 15349082

[37] Isojärvi J. Disorders of reproduction in patients with epilepsy: antiepileptic drug related mechanisms. Seizure. 2008 3;17(2):111–9. 10.1016/j.seizure.2007.11.007 18164216

[38] Otoom S, Batieneh H, Hassan Z, Daoud A. Effects of long-term use Topiramate on fertility and growth parameter in adult male rats. Neuro Endocrinol Lett. 2004 10;25(5):351–5. 15580169

[39] US EPA [Internet]. Guidelines for Reproductive Toxicity Risk Assessment EPA/630/R-96/009 dated October 1996 [cited 2015 May 27]. http://www2.epa.gov/sites/production/files/201411/documents/guidelines_repro_toxicity.pdf

[40] Wisniewski P, Romano RM, Kizys MM, Oliveira KC, Kasamatsu T, Giannocco G et al Adult exposure to bisphenol A (BPA) in Wistar rats reduces sperm quality with disruption of the hypothalamic-pituitary-testicular axis. Toxicology. 2015 3 2;329:1–9. 10.1016/j.tox.2015.01.002 25575453

[41] Lin JF, Lin YH, Liao PC, Lin YC, Tsai TF, Chou KY et al Induction of testicular damage by daily methamphetamine administration in rats. Chin J Physiol. 2014 2 28;57(1):19–30. 10.4077/CJP.2014.BAB155 24621335

[42] Andretta RR, Okada FK, Paccola CC, Stumpp T, de Oliva SU, Miraglia SM. Carbamazepine-exposure during gestation and lactation affects pubertal onset and spermatic parameters in male pubertal offspring. Reprod Toxicol. 2014 4;44:52–62. 10.1016/j.reprotox.2013.09.009 24126190

[43] Kumar S, Kaur G. Second generation anti-epileptic drugs adversely affect reproductive functions in young non-epileptic female rats. Eur Neuropsychopharmacol. 2014 10;24(10):1709–18. 10.1016/j.euroneuro.2014.06.011 25213092

[44] Svalheim S, Taubøll E, Luef G, Lossius A, Rauchenzauner M, Sandvand F et al Differential effects of levetiracetam, carbamazepine, and lamotrigine on reproductive endocrine function in adults. Epilepsy Behav. 2009 10;16(2):281–7. 10.1016/j.yebeh.2009.07.033 19716343

[45] Taubøll E, Gregoraszczuk EL, Wojtowicz AK, Milewicz T. Effects of levetiracetam and valproate on reproductive endocrine function studied in human ovarian follicular cells. Epilepsia. 2009 8;50(8):1868–74. 10.1111/j.1528-1167.2009.02131.x 19490055

[46] Harden CL, Nikolov BG, Kandula P, Labar DR, Pannullo S. Effect of levetiracetam on testosterone levels in male patients. Epilepsia. 2010 11;51(11):2348–51. 10.1111/j.1528-1167.2010.02732.x 21175608

[47] Rauchenzauner M, Bitsche G, Svalheim S, Taubøll E, Haberlandt E, Wildt L et al Effects of levetiracetam and valproic acid monotherapy on sex-steroid hormones in prepubertal children—results from a pilot study. Epilepsy Res. 2010 2;88(2–3):264–8. 10.1016/j.eplepsyres.2009.11.003 20015617

[48] Calabrò RS, Bramanti P. Levetiracetam-induced sexual disorders. Seizure. 2013 5;22(4):329 10.1016/j.seizure.2013.01.003 23340273

[49] Csoka AB, Shipko S. Persistent sexual side effects after SSRI discontinuation. Psychother Psychosom. 2006;75(3):187–8. 10.1159/000091777 16636635

[50] Fanaei H, Khayat S, Halvaei I, Ramezani V, Azizi Y, Kasaeian A et al Effects of ascorbic acid on sperm motility, viability, acrosome reaction and DNA integrity in teratozoospermic samples. Iran J Reprod Med. 2014 2;12(2):103–10. 24799867 PMC4009562

[51] Giribabu N, Kumar KE, Rekha SS, Muniandy S, Salleh N. *Chlorophytum borivilianum* (Safed Musli) root extract prevents impairment in characteristics and elevation of oxidative stress in sperm of streptozotocin-induced adult male diabetic Wistar rats. BMC Complement Altern Med. 2014 8 8;14:291 10.1186/1472-6882-14-291 25104050 PMC4141081

[52] Lewis SE, John Aitken R, Conner SJ, Iuliis GD, Evenson DP, Henkel R et al The impact of sperm DNA damage in assisted conception and beyond: recent advances in diagnosis and treatment. Reprod Biomed Online. 2013 10;27(4):325–37. 10.1016/j.rbmo.2013.06.014 23948450

[53] Abdel-Wahab BA, Shaikh IA, Khateeb MM, Habeeb SM. Omega 3 polyunsaturated fatty acids enhance the protective effect of levetiracetam against seizures, cognitive impairment and hippocampal oxidative DNA damage in young kindled rats. Pharmacol Biochem Behav. 2015 8;135:105–13. 10.1016/j.pbb.2015.05.020 26044965

[54] Marini H, Costa C, Passaniti M, Esposito M, Campo GM, Ientile R et al Levetiracetam protects against kainic acid-induced toxicity. Life Sci. 2004 1 23;74(10):1253–64. 14697408 10.1016/j.lfs.2003.08.006

[55] Mazhar F, Malhia SM, Simjeea SU. Comparative studies on the effects of clinically used anticonvulsants on the oxidative stress biomarkers in PTZ-induced kindling model of epileptogenesis in mice, 11th Annual Focused Issue on Methods in Safety Pharmacology, J Pharmacol Toxicol Methods. 2014 Nov-Dec;70(3):313. 10.1515/jbcpp-2016-0034 27658141

[56] Oliveira AA, Almeida JP, Freitas RM, Nascimento VS, Aguia LM, Junior HV, et al Effects of levetiracetam in lipid peroxidation level, nitrite-nitrate formation and antioxidant enzymatic activity in mice brain after pilocarpine-induced seizures. Cell Mol Neurobiol. 2007 1;27(3):395–406. 10.1007/s10571-006-9132-y 17205390 PMC11881810

[57] Ueda Y, Doi T, Takaki M, Nagatomo K, Nakajima A, Willmore LJ. Levetiracetam enhances endogenous antioxidant in the hippocampus of rats: in vivo evaluation by brain microdialysis combined with ESR spectroscopy. Brain Res. 2009 4 17;1266:1–7. 10.1016/j.brainres.2009.02.040 19268434

[58] Sarangi SC, Kakkar AK, Kumar R, Gupta YK. Effect of lamotrigine, levetiracetam and topiramate on neurobehavioral parameters and oxidative stress in comparison with valproate in rats, 10^th^ Annual Focused Issue on Methods in Safety Pharmacology, J Pharmacol Toxicol Methods. 2013 Jul-Aug;68(1):e4. 10.4103/0971-5916.193296 PMC5116881 27834333

[59] Varoglu AO, Yildirim A, Aygul R, Gundogdu OL, Sahin YN. Effects of valproate, carbamazepine, and levetiracetam on the antioxidant and oxidant systems in epileptic patients and their clinical importance. Clin Neuropharmacol. 2010 5;33(3):155–7. 10.1097/WNF.0b013e3181d1e133 20502135

[60] Khan S, Jan MH, Kumar D, Telang AG. Firpronil induced spermotoxicity is associated with oxidative stress, DNA damage and apoptosis in male rats. Pestic Biochem Physiol. 2015 10;124:8–14. 10.1016/j.pestbp.2015.03.010 26453224

[61] Chi HJ, Chung DY, Choi SY, Kim JH, Kim GY, Lee JS et al Integrity of human sperm DNA assessed by the neutral comet assay and its relationship to semen parameters and clinical outcomes for the IVF-ET program. Clin Exp Reprod Med. 2011 3;38(1):10–7. 10.5653/cerm.2011.38.1.10 22384412 PMC3283044

[62] Knopper LD. Use of The Comet Assay to Assess Genotoxicity In Mammalian, Avian, and Amphibian Species, Technical Report Series No. 429, Canadian Wildlife Service, 2005.

[63] Møller P, Loft S, Ersson C, Koppen G, Dusinska M, Collins A. On the search for an intelligible comet assay descriptor. Front Genet. 2014 7 17;5:217 10.3389/fgene.2014.00217 25101109 PMC4101262

[64] Hauser R, Singh NP, Chen Z, Pothier L, Altshul L. Lack of an association between environmental exposure to polychlorinated biphenyls and p,p'-DDE and DNA damage in human sperm measured using the neutral comet assay. Hum Reprod. 2003 12;18(12):2525–33. 14645167 10.1093/humrep/deg508

[65] Morris ID, Ilott S, Dixon L, Brison DR. The spectrum of DNA damage in human sperm assessed by single cell gel electrophoresis (Comet assay) and its relationship to fertilization and embryo development. Hum Reprod. 2002 4;17(4):990–8. 11925396 10.1093/humrep/17.4.990

[66] Olive PL, Banáth JP. The comet assay: a method to measure DNA damage in individual cells. Nat Protoc. 2006;1(1):23–9. 10.1038/nprot.2006.5 17406208

[67] Rasool EA, Abdul-Rasheed OF. AL-Hashimi AF. Comparison Between Different DNA and Conventional sperm parameters in Infertile Men, Al-kindy Col Med J. 2012;8(2):40–7.

[68] El-Shorbagy HM, Hamdi H. Genotoxic and Mutagenic Studies of The Antiepileptic Drug Levetiracetam in Pregnant Rats and Their Fetuses. Int J Pharm Sci. 2016;8(2),82–8.

[69] Shi JQ, Wang BR, Tian YY, Xu J, Gao L, Zhao SL et al Antiepileptics topiramate and levetiracetam alleviate behavioral deficits and reduce neuropathology in APPswe/PS1dE9 transgenic mice. CNS Neurosci Ther. 2013 11;19(11):871–81. 10.1111/cns.12144 23889921 PMC6493595

[70] Stettner M, Dehmel T, Mausberg AK, Köhne A, Rose CR, Kieseier BC. Levetiracetam exhibits protective properties on rat Schwann cells in vitro. J Peripher Nerv Syst. 2011 9;16(3):250–60. 10.1111/j.1529-8027.2011.00355.x 22003940

